# Traditional, modern or mixed? Perspectives on social, economic, and health impacts of evolving food retail in Thailand

**DOI:** 10.1007/s10460-014-9561-z

**Published:** 2014-11-11

**Authors:** Matthew Kelly, Sam-ang Seubsman, Cathy Banwell, Jane Dixon, Adrian Sleigh

**Affiliations:** 1 National Centre for Epidemiology and Population Health, Research School of Population Health, The Australian National University, Canberra, ACT 0200 Australia; 2 School of Human Ecology, Sukhothai Thammathirat Open University, Nonthaburi, Thailand

**Keywords:** Food retail, Thailand, Food culture, Fresh markets, Transnational supermarkets, Consumer perspectives

## Abstract

Transnational food retailers expanded to middle-income countries over recent decades responding to supply (liberalized foreign investment) and demand (rising incomes, urbanization, female workforce participation, and time poverty). Control in new markets diffuses along three axes: socio-economic (rich to poor), geographic (urban to rural), and product category (processed foods to fresh foods). We used a mixed method approach to study the progression of modern retail in Thailand on these three axes and consumer preferences for food retailing. In Thailand modern retail controls half the food sales but traditional fresh markets remain important. Quantitative questionnaires administered to members of a large national cohort study revealed around half of respondents were primarily traditional shoppers and half either utilized modern and traditional formats equally or primarily shopped at supermarkets. Fresh foods were mainly purchased at traditional retail formats and dry packaged foods at supermarkets. Qualitative interviews found price and quality of produce and availability of culturally important products to be significant reasons for continued support of fresh markets. Our results show socio-economic and geographic diffusion is already advanced with most respondents having access to and utilizing modern retail. Control of the fresh food sector by transnationals faces barriers in Thailand and may remain elusive. The short to mid-term outcome may be a bifurcated food system with modern and traditional retail each retaining market share, but fresh markets longer term survival may require government assistance as supermarkets become more established. Fresh markets supply affordable, healthy foods, and livelihoods for poorer Thais and are repositories of Thai food culture and social networks. If they survive they will confer cultural, social, economic, and health benefits.

## The evolution of food retailing: first the west then the rest

In most high-income economies the food retail sector is dominated by modern food retail formats—hypermarkets, supermarkets, and convenience stores. Hypermarkets sell foods and virtually all other goods, supermarkets specialize in processed and fresh foods, and convenience stores are smaller format and focus on snack foods. These retail systems first evolved in the USA in the 1930s; after World War II they spread to most industrialized countries including North America, Europe, and Australia. Their increased coverage was assisted by the suburban movement of population, the growth of the industrialized food processing and packaging industry, and other social changes, particularly increasing incomes and women’s participation in the workforce (Stiegert and Kim 2009; Goodman and Redclift 1991; Banwell et al. 2012). Supermarket control was first exerted for processed and packaged food but eventually led to domination of the fresh food sector. In the USA it took 40 years for supermarkets to gain control of the fresh food sector (Reardon et al. 2012) but in other countries this control is still contested by other fresh food outlets.

More recently supermarket expansion has been into low and middle-income developing countries (LMICs), beginning in Latin America in the early 1990s and proceeding to Asia in the next decade (Reardon and Berdegue 2006; Reardon et al. 2012). As in high income countries supermarket demand was driven by rising incomes, urbanization, female workforce participation, growing reliance on cars, and time-poor consumers. Increasingly globalized media and international travel also increased interest in novel and “global foods” (Traill 2006). The growth of free trade agreements and the continued liberalization of foreign direct investment laws through the 1990s drove the supply side which enabled these changes in consumer demand to be realized. By the 1990s modern food retail control of markets in high-income countries had reached saturation. However, liberalization of trade and investment allowed European and North American food retailers to continue expanding (Hawkes 2005). The speed of change has been noteworthy; what took 60 years in the developed world has been compressed into just two decades in Latin America and countries first affected in Asia, and modern food retail expansion now appears to be occurring even faster in China and Vietnam (Reardon et al. 2012).

In this paper we investigate Thailand as a case study of modern retail diffusion in a fast developing middle-income country. Thailand has experienced a rapid expansion of supermarkets, hypermarkets and convenience stores in the last two decades but still retains a dynamic and culturally valued traditional fresh market sector. We first present an overview of theories on the diffusion of modern retail, followed by an examination of food retailing trends in Thailand. We then present the results of our mixed method study into consumer utilization of various forms of food retail format and consumer food shopping preferences and motivations. Our research questions address how far Thailand has progressed towards modern retail formats, the social-geography of these trends and the prospects for continued growth of supermarkets, and survival of fresh markets. We pay particular attention to differences in shopping patterns between fresh foods and packaged processed foods. With fresh markets currently functioning as affordable sources of health promoting fresh foods and sources of livelihoods for some of Thailand’s poorest groups, the results of this study have implications for equitable social, economic, and health development in modern Thailand.

## Literature review

### Theory of retail diffusion

The spread of supermarkets into LMICs has been observed by Reardon and Berdegue (2006) to proceed along three axes of diffusion, in sequence or concurrently in different settings. These axes (and diffusion direction) relate to wealth (rich to poor), urbanization (urban to rural), and food category (processed to fresh) and are summarized as follows.

(1) Socio-economic diffusion. In LMICs modern food retailers begin by targeting high and middle-income consumers who have disposable income, private transport, high opportunity costs, and more exposure to modern, novel foods. Later modern food retailers diversify their products offering low cost mass-produced packaged and processed foods that appeal to low-income consumers. Hypermarkets and superstores then emerge offering lower prices with less emphasis on appearance, modernity, and prestige.

(2) Geographic diffusion. In LMICs modern food retailers tend to first establish in high-income urban areas. Once established, they diffuse into regional centers and rural areas. Rising rural incomes and increasing exposure to global food products in many countries coupled with a lack of rural food retail services mean this geographic spread is ongoing and successful in many Asian settings.

(3) Product category diffusion. This part of the diffusion process is the most contested and the most challenging for modern food retail companies. Initially, the biggest competitive advantage enjoyed by supermarkets is in processed packaged foods. They are cheaper to produce and novel foods in developing markets. Fresh produce sectors including meat, fruit, and vegetables have been more difficult for modern food retailers to dominate. There are several obstacles including: difficulty for modern food retailers to achieve reliable price-stable procurement chains; perceptions of inferior freshness and quality of fruit and vegetables sold at supermarkets; and cultural and social values associated with fresh markets.

As noted earlier, in high-income countries there was a substantial lag between the establishment of supermarkets and their move into the fresh food sector. It is therefore not surprising that in countries where modern food retailing has only been significant for 10–15 years traditional markets still largely control fresh food sales. However, there are indications that supermarket control of the fresh food sector is already underway in those LMICs which first experienced the expansion of modern retail in the early 1990s (Latin America). Nevertheless, some observers contend that enduring competitive advantages ensure that traditional fresh food retailers (particularly in Asia) will impede supermarket penetration of fresh food sales (Goldman et al. 2002).

### A case study: transformations in the food retail sector in Thailand

The food retail sector in Thailand has until recently been dominated by two types of stores, the fresh market and small family run general stores. Thailand’s retail sector began to evolve in the 1960s with the establishment of the country’s first supermarkets and department stores which catered to high-income Thai and expatriate consumers (Wigglesworth and Brotan 1966). Substantial change did not occur however until the 1980s, with an economic boom in Thailand. Rising incomes and the expansion of Bangkok into “suburban areas” meant increased car ownership and dependence as well as an increasing demand for convenient one-stop shopping and a growing interest in western style foods and lifestyles. Through the 1990s supermarket numbers expanded to meet this demand as well as further retail diversification including the introduction of 7-Eleven convenience stores in 1989 (Tokrisna 2007). In accordance with theories of modern retail diffusion proposed by Reardon and others and described above, the initial expansion of modern retail in Thailand took place primarily in Bangkok (Schaffner et al. 2005).

The Asian financial crisis of 1997 was a catalyst for widespread change in the Thai food retailing sector. Much of modern food retailing until that point had been controlled by Thai capital or Thai-foreign partnerships. Beginning in 1997 many Thai retail firms were dissolved or taken over by foreign partners. The Thai government also relaxed foreign investment laws allowing an influx of transnational food retailers who now dominate modern food retail in Thailand. These transnational food companies, including Tesco (UK), Carrefour, and Big C (French), proceeded to massively expand their operations (Schaffner et al. 2005).

Led by transnational companies socio-economic and geographic diffusion of modern food retail proceeded. Socio-economic diffusion was evident in the formats of modern food retail that became prevalent from the late 1990s. Instead of the supermarkets within department stores described above, large format hypermarkets and small format convenience stores experienced the biggest growth. These new formats no longer marketed themselves to high-income groups but instead aimed to use low prices and range of products to attract middle and lower class consumers (Shannon 2009). Convenience stores were particularly successful in this task in many urban areas largely taking the place of traditional family run general stores that had fulfilled a similar role for small, daily purchases. Geographic diffusion from 2001 saw modern retail outlets opening in many regional centers and even in more rural areas (Phongpaichit and Baker 2007).

In the mid-1980s, 95 % of food retailing in Thailand had been in the traditional sector. Only 20 years later around half of food retail was controlled by modern food retail outlets (Kuipers 2007; Tokrisna 2007), and some estimates now put that figure at nearly 70 % in 2012 (Global Agricultural Information Network 2013). In 1997 there were 50 supermarkets and 60 hypermarkets in the nation; by 2007 corresponding numbers were 166 and 225. The number of convenience stores expanded from 1,180 to 6,263 and by 2011 had reached nearly 12,000 outlets (Global Agricultural Information Network 2013). The difficulty of obtaining detailed sales data from the traditional sector, where trade is still substantially informal and cash based, means making conclusions in more detail particularly regarding food category diffusion is problematic. However, significant growth of hypermarkets and convenience stores indicates modern food retail diffusion has occurred across socio-economic groups.

The outcomes of this process of modern food retail diffusion and the future mix of food retail formats have implications for social, economic, and health equity in Thailand. Several studies have found fresh markets in Thailand are significantly cheaper sources of health promoting fresh foods when compared with supermarkets (Isaacs 2009; Schaffner et al. 2005). Any reduction in fresh markets may have negative impacts on the ability of poor Thai consumers to access healthy foods (Kelly et al. 2010). Also, supermarkets, and transnational food companies more generally, have been observed to increase the availability of “problem foods” which are low cost, energy dense, nutrient poor and highly processed. These foods are linked to negative health outcomes (obesity, diabetes, cardiovascular disease, and some cancers) of the nutrition transition that accompanies socio-economic development in most LMICs (Hawkes 2005, 2008). As well as these nutrition benefits, fresh markets also provide a source of livelihood for many thousands of lower income Thais. These market traders derive not only financial support but also social capital and the health and social benefits provided by closely connected friendship, kinship, and commercial relationships (Banwell et al. 2013).

## Methods and procedures

**Fig. 1:**
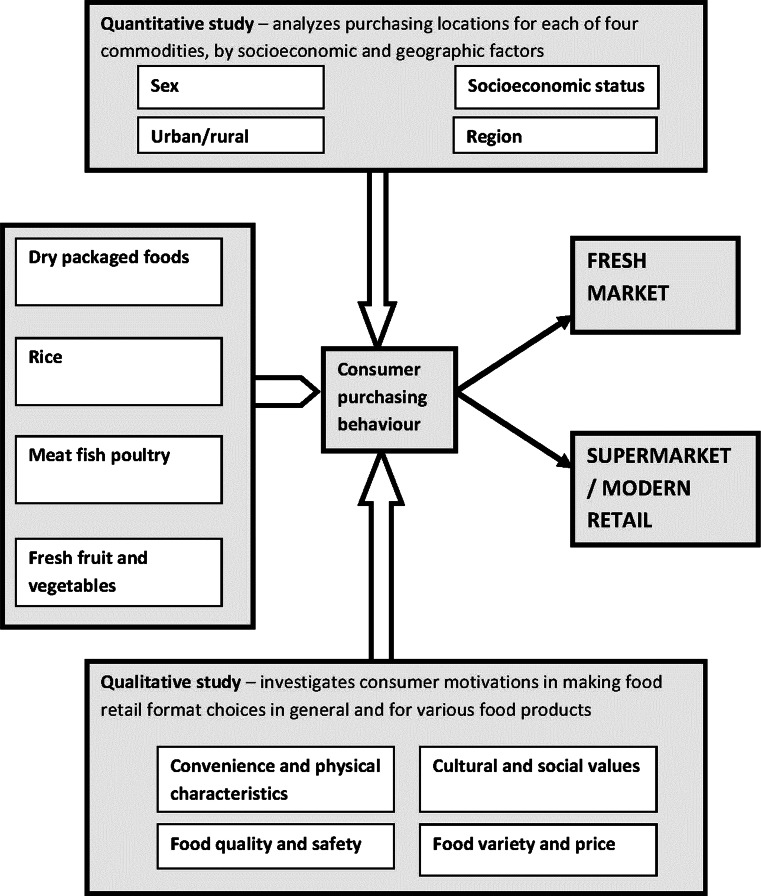
Conceptual framework for mixed method study quantifying retail usage and its determinants and analyzing consumer attitudes and motivations

This study uses a mixed method approach guided by our conceptual framework (Fig. 1), with questionnaires collecting quantitative data and in-depth interviews providing qualitative insights. Combining the two methods makes it possible to gain a more comprehensive understanding of Thai consumer behavior and motivations. Quantitative data allowed statistical analysis of shopping patterns and how they differed among socio-economic and geographic groups while interviews allowed us to understand in greater depth consumer decision making considerations as well as reasons for food retail format choice preferences.

### Study population

In 2005 a large nationwide study of the health-risk transition underway in Thailand was begun with detailed questionnaires being mailed to all 200,000 students then enrolled at Sukhothai Thammathirat Open University (STOU), a distance education institution. The 87,134 respondents to this survey formed the baseline cohort for our study, the Thai Cohort Study. The questionnaire focused on pre- and post-transition health outcomes (infections, injuries, and chronic diseases) and health-risks (diet, behavior, and socio-economic status). Information was collected on a wide array of socio-economic, demographic, and personal characteristics of respondents. At baseline (2005) the cohort members were aged from 15 to 87, mean age 29, lived in all regions of Thailand and like the general Thai population on average were of modest financial means. Details on cohort recruitment have been reported elsewhere (Seubsman et al. 2011; Sleigh et al. 2008). In 2009 a four-year follow-up questionnaire was conducted with 60,569 responding (70 %).

In 2012, a sub-sample of 3,400 Thai Cohort Study members was sent an additional questionnaire focused on local food retail environments, food provisioning patterns, and food retail preferences. This sub-sample was postcode defined and included all cohort members in one major urban area and surrounding hinterland, in each region of Thailand. Areas included were Nonthaburi and Central Bangkok (Central region), Khon Kaen (Northeast region), Nakhon Sri Thammarat (Southern region), and Chiang Mai (Northern region). These locations ensured representation of each region of Thailand, as well as including areas with higher (Bangkok) and lower (Nakhon Sri Thammarat) levels of modern retail penetration. We chose a predominantly urban sample in each region as these would be a group who had more modern retail access. These four areas were also sites where previous field research had been conducted and thus were areas with which the researchers were familiar (Banwell et al. 2013). The questionnaire was answered by 1,516 cohort members (45 %), a responding sub-sample that forms the study population for this current food environment and food provisioning research.

### Questionnaire measures and definitions

Personal characteristics of respondents used in this analysis include urban or rural residence over the lifecourse (in 2012, in 2005 and when a child aged 10–12 years old), region of residence (Bangkok and Central Thailand, North, Northeast, and Southern Thailand), age (<45 years and ≥45 years, chosen because mean age of sample was 42.5 years), income (<30,000 baht per month and ≥30,000 baht per month, chosen because 30,000 baht was the average monthly income for the sample and represents an average Thai urban household income), work hours per week (<20, 20–40, and >40 h), household size (1–4, 5–9, and ≥10 persons), and household vehicle ownership (bicycle only, motorcycle only, car only and multiple vehicle ownership).

Food retail environments and food shopping behavior were assessed. Firstly, participants were asked whether their “local area” (subjectively defined) contained food outlets that were *traditional* (fresh market, general store or mobile vendor), and/or *modern* (convenience store or supermarket). Approximate travel times to the closest of each of these types of food store were recorded. The frequency of visiting each food store type was measured on a four-point scale (never/less than monthly, 1–3 times per month, 1–2 times per week, and daily or more). Relative shopping frequencies (combined fresh market and general store versus combined supermarket and convenience store) were then used to classify respondents’ choice of venue as “mainly traditional,” “mainly modern,” or “mixed” (i.e., equal). Respondents were also asked where they would normally purchase the following staple food items: rice; animal protein (meat/fish/poultry); fresh fruit and vegetables; snacks/sweets; and dried packaged foods. Also, the average weekly spend on each of these food categories was reported.

As well, a set of questions measured the perceived importance of an array of factors when deciding where to purchase food. These factors were affordability, travel convenience, car parking, hygiene-food safety, service, variety, availability of local/traditional foods, store attractiveness, promotions- sales-coupons, and healthy foods. Participants were asked to rate the importance of each of these factors on a scale of 0 (unimportant) to 10 (very important).

### Analysis of questionnaires

Completed questionnaires were scanned and digitized by Scandevet intelligent character recognition software developed by computer programmers at Khon Kaen University. Further data verification, correction, and editing were completed using My SQL software and for analysis we used SPSS (Version 20). Proportions were compared using Chi square tests with significant *p* values set at 5 %.

For each of the groups defined by relative shopping frequency (“traditional,” “modern,” and “mixed”), the prevalence of socio-economic and demographic attributes were compared. Favored locations (“traditional” *versus* “modern”) for purchase of key food types were tabulated by socio-economic, geographic, and demographic characteristics. We then constructed logistic regression models to test associations between socio-economic-geographic factors (independent variables) and location of purchase of rice, meat/fish/chicken, fresh fruit and vegetables, and dried/instant/packaged foods (dependent variables). We present logistic regression coefficients and standard errors, adjusted odds ratios and 95 % CIs for these relationships adjusting for income, urban/rural residence, region of residence, age, and sex. These variables were adjusted for in the multi-variate model as they were shown on bivariate analysis to be significantly associated with particular shopping patterns. Model performance was assessed by the c-statistic to measure discrimination and with the Hosmer and Lemeshow goodness-of-fit test (Hosmer and Lemeshow 1989, p. 140). For perceived importance of an array of factors potentially influencing food-purchasing venue (rated 0–10, see above) mean scores were calculated for food shopping groups (“traditional,” “modern,” and “mixed”) as well as by socio-economic and geo-demographic characteristics.

### Qualitative data and analysis

After questionnaire responses were received and analyzed, a purposive age-sex-income balanced sample of respondents was selected for in-depth interviews in Chiang Mai (n = 8) and Nonthaburi-Bangkok (n = 8). Respondents in each setting were divided into 8 groups by sex, income (lower vs. higher) and age (<40 and ≥40). Interviews began with questions on respondent households and their lifetime residence/migration history and then proceeded to more open-ended questions on their food shopping patterns, attitudes towards various food retail formats, and views on future trends in food retailing in Thailand. The sample size was based on recent research indicating that for purposive non-probabilistic samples, little new information is gained after six to 12 interviews (Guest et al. 2006).

Interviews were conducted in various settings considered most convenient for the interviewees and included participants’ homes, workplaces, public parks, food courts, and at the researcher’s University campus (STOU). The lead author conducted the interviews in Thai with a skilled Thai research assistant. All interviews were recorded and transcribed by both interviewers and the lead author then translated key results into English. The lead author coded and analyzed the interviews building a set of themes corresponding to the key study questions. Recurring cultural influences were noted and explanations for attitudes towards food retail formats extracted and summarized.

## Results

### Questionnaires: shopping patterns

The sample was 56.4 % female and predominantly urban (74.9 %), though 17 % of the sample had moved to an urban area in the last 8 years (Table 1). Just under half of participants lived in Bangkok and Central Thailand with the remainder fairly evenly divided between the other regions of Thailand. The majority lived in households of four or fewer people and 66.3 % were aged less than 45 years. Overall, around half the sample was classified as primarily fresh market shoppers, nearly a third mainly shopped at modern retail outlets, and the remainder utilized both retail formats equally. However, these figures measure relative frequency of visiting each store type, and most consumers did utilize modern and traditional retail with only 17 % reporting never visiting supermarkets and 13 % never visiting fresh markets. Rural participants and those who had only moved to urban areas since 2005 were significantly more likely to be fresh market shoppers than longer term urban residents (60.3, 52.7, and 50.7 % respectively). Northern Thailand had the highest proportion of fresh market shoppers and Bangkok and the Central Region the lowest (66.7 vs. 44.3 %). Older people (54.8 vs. 52.5 %), those with lower incomes (56.1 vs. 50.3 %), and those living in larger households (56.5 and 54.5 vs. 52.3 %) were also more likely to be fresh market shoppers. Household vehicle ownership also had a strong relationship with shopping pattern with those owning a bicycle or motorcycle only significantly more likely to be mainly fresh market shoppers than car owners (65, 60, and 47 % respectively).

**Table 1:** Food provisioning patterns by socio-demographic and personal characteristics

	Predominant shopping venue*			
	Fresh market shoppers	Mixed shoppers	Supermarket shoppers	Total
	N (%)**	N (%)**	N (%)**	N (%)***
Overall	808 (53.3)	286 (18.9)	423 (27.9)	
Male	349 (52.8)	138 (20.9)	174 (26.3)	661 (43.6)
Female	458 (53.6)	148 (17.3)	249 (29.1)	855 (56.4)
Residence				
Rural	226 (60.3)^†^	75 (20.0)	74 (19.7)^†^	375 (25.1)
Urban	568 (50.7)	208 (18.6)	344 (30.7)	1120 (74.9)
Persistent rural^a^	180 (62.1)	54 (18.6)	56 (19.3)	290 (19.1)
Newly urban^b^	97 (52.7)	33 (17.9)	54 (29.3)	184 (12.3)
Long-term urban^c^	463 (50.2)	173 (18.7)	287 (31.1)	923 (60.1)
De-urbanisers^d^	44 (53.7)	20 (24.4)	18 (22.0)	82 (5.4)
Region				
Bangkok and central	306 (44.3)^†^	137 (19.8)	248 (35.9)^†^	691 (46.1)
North	202 (66.7)	53 (17.5)	48 (15.8)	303 (20.2)
Northeast	178 (56.9)	55 (17.6)	80 (25.6)	313 (20.9)
South	121 (57.9)	41 (19.6)	47 (22.5)	209 (13.9)
Age				
<45	522 (52.5)	194 (19.5)	279 (28.0)	995 (66.3)
45+	278 (54.8)	87 (17.2)	142 (28.0)	507 (33.7)
Income				
Low	434 (56.1)^†^	156 (20.2)	184 (23.8)^†^	774 (51.6)
High	366 (50.3)	125 (17.2)	237 (32.6)	728 (48.4)
Work hours				
<20	187 (52.1)	71 (19.8)	101 (28.1)	359 (23.9)
20–40	294 (54.4)	100 (18.5)	146 (27.0)	540 (36.0)
41+	295 (52.0)	106 (18.7)	166 (29.3)	567 (37.8)
Household size				
1–4	582 (52.3)	213 (19.2)	317 (28.5)	1,112 (74.1)
5–9	210 (56.5)	63 (16.9)	99 (26.6)	372 (24.8)
10+	12 (54.5)	5 (22.7)	5 (22.7)	22 (1.4)
Vehicle ownership				
Bicycle only	17 (65.4)^†^	3 (11.5)**	6 (23.1)^†^	26 (1.7)
Motorcycle only	72 (60.0)	27 (22.5)	21 (17.5)	120 (7.9)
Car only	109 (47.0)	36 (15.5)	87(37.5)	232 (15.3)
Multiple vehicles owned	448 (54.0)	157 (18.9)	224 (27.0)	829 (54.7)

^a^Rural in 2005 and now

^b^Rural in 2005, urban now

^c^Urban in 2005 and now

^d^Urban in 2005, rural now

* Predominant shopping venue = retail format visited most often; if supermarket and traditional were equally frequent respondent is classified as “mixed shopper”

** Row percentage (% of each group who are fresh, mixed or modern shoppers)

*** Column percentage (% of whole sample belonging to each category)

^† ^Difference between column proportions significant with *p* < 0.001

Rice (Table 2) was purchased by just over half of respondents at modern retail formats but this figure fell to only 38 % among rural residents. Northern Thais were most likely to buy rice at a traditional retail format and Bangkok and Central residents most likely to buy at a modern outlet. Lower income participants were also significantly more likely to purchase rice at a traditional format store. For meat/fish/chicken (Table 3) and fresh fruit and vegetables (Table 4), traditional formats were much more likely to be utilized (80.8 and 86.4 % respectively). Shopping patterns for these food categories also differed by region, rural–urban residence, and income, with rural, non-Bangkok, and lower income participants all significantly more likely to buy meat, fruit, and vegetables from traditional format stores. More Southern Thai participants bought meat (87.0 %) and fruit and vegetables (91.5 %) from traditional outlets. Most (87 %) participants purchased dried, packaged, and instant foods (Table 5), from modern retail outlets. Rural residents, Northeastern Thailand residents, and lower income participants were significantly more likely to buy these foods from a traditional outlet with the largest proportion of traditional shoppers found among rural residents (22.5 %).

**Table 2:** Rice purchasing location by socio-demographic and personal characteristics

	Traditional outlets		Modern outlets	
	n	%	n	%
Overall	596	43.5	775	56.5
Residence				
Rural	198	61.9*	122	38.1*
Urban	386	37.4	647	62.6
Region				
Bangkok and central	206	31.5*	447	68.5*
North	162	58.9	113	41.1
Northeast	122	49.4	125	50.6
South	105	53.8	90	46.2
Age (years)				
<45	401	44.5	503	55.6
≥45	187	41.2	267	58.8
Monthly Income				
<30,000 baht	356	51.2*	339	48.8*
≥30,000 baht	236	35.5	428	64.5

Not all respondents answered every question so totals for each analysis may vary

* Significant (*p* < 0.0001) difference between groups, e.g., rural versus urban

**Table 3:** Meat fish chicken purchasing location by socio-demographic and personal characteristics

	Traditional outlets		Modern outlets	
	n	%	n	%
Overall	1,084	80.8	348	19.2
Residence				
Rural	302	84.8*	51	15.2*
Urban	768	72.4	293	27.6
Region				
Bangkok and central	450	69.3*	199	30.7*
North	222	77.6	64	22.4
Northeast	237	80.0	59	20.0
South	174	87.0	26	13.0
Age (years)				
<45	729	76.8	220	23.2
≥45	345	73.2	126	26.8
Monthly Income				
<30,000 baht	596	81.8*	133	18.2*
≥30,000 baht	482	69.7	210	30.3

Not all respondents answered every question so totals for each analysis may vary

* Significant (*p* < 0.0001) difference between groups, e.g., rural versus urban

**Table 4:** Fruit and vegetable purchasing location by socio-demographic and personal characteristics

	Traditional outlets		Modern outlets	
	n	%	n	%
Overall	1,230	86.4	194	13.6
Residence				
Rural	330	92.2*	28	7.8*
Urban	884	84.4	164	15.6
Region				
Bangkok and central	544	83.8*	105	16.2*
North	248	87.3	36	12.7
Northeast	255	87.6	36	12.4
South	182	91.5	17	8.5
Age (years)				
<45	828	87.2	121	12.8
≥45	391	84.4	72	15.6
Monthly Income				
<30,000 baht	662	91.1*	65	8.9*
≥ 30,000 baht	562	81.9	124	18.1

Not all respondents answered every question so totals for each analysis may vary

* Significant (*p* < 0.0001) difference between groups, e.g., rural versus urban

**Table 5:** Dried packaged instant foods purchasing location by socio-demographic and personal characteristics

	Traditional outlets		Modern outlets	
	n	%	n	%
Overall	175	12.6	1,209	87.4
Residence				
Rural	79	22.8*	268	77.2*
Urban	91	8.9	929	91.1
Region				
Bangkok and central	88	13.3*	576	86.7*
North	34	12.3	242	87.7
Northeast	54	19.4	224	80.6
South	29	14.9	166	85.1
Age (years)				
<45	106	11.6	810	88.4
≥45	65	14.2	392	85.8
Monthly Income				
<30,000 baht	115	16.3*	589	83.7*
≥30,000 baht	58	8.7	610	91.3

Not all respondents answered every question so totals for each analysis may vary

* Significant (*p* < 0.0001) difference between groups, e.g., rural versus urban

Table 6 presents logistic regression models between various socio-economic-geographic factors and purchasing location of key food types. For each of the four models constructed (for rice, meat products, fresh produce, and dry packaged foods) discrimination was reasonable, with c-statistics ranging from 0.63 to 0.68. Hosmer–Lemeshow tests showed no significant lack of fit (*p* values range from 0.1 to 0.85). The models presented show clear and significant associations between urban residence, higher income, residing in Bangkok, and purchasing all food types at modern food retailers. The strongest relationships were for urban residence and dry foods purchase (B = 0.91, AOR 2.47), for high income and fresh food purchase (B = 0.72, AOR 2.05), and for Bangkok residence and rice purchase (B = 0.76, AOR 2.13).

**Table 6:** Logistic regression models measuring associations between socio-demographic factors and modern retail use for four main food types

	Rice		Meat, fish, chicken		Fresh fruit and vegetables		Dry, packaged, and instant foods	
	B (SE)	AOR^#^ (95 % CI)	B (SE)	AOR^#^ (95 % CI)	B (SE)	AOR^#^ (95 % CI)	B (SE)	AOR^#^ (95 %CI)
Region								
Outside Bangkok	Ref	1	Ref	1	Ref	1	Ref	1
Bangkok	0.76 (0.12)	2.13 (1.68–2.71)**	0.42 (0.14)	1.52 (1.17–1.98)**	0.19 (0.17)	1.20 (0.87–1.67)	0.22 (0.19)	1.24 (0.85–1.81)
Residence								
Rural	Ref	1	Ref	1	Ref	1	Ref	1
Urban	0.66 (0.14)	1.94 (1.46–2.56)**	0.57 (0.18)	1.76 (1.24–2.50) **	0.61 (0.23)	1.84 (1.20–3.02)**	0.91 (0.19)	2.47 (1.71–2.58)**
Income								
Lower	Ref	1	Ref	1	Ref	1	Ref	1
Higher	0.56 (0.12)	1.75 (1.38–2.21)**	0.58 (0.13)	1.79 (1.38–2.32)**	0.72 (0.17)	2.05 (1.49–2.90)**	0.65 (0.18)	1.92 (1.34–2.75)**
Age								
<45 years	Ref	1	Ref	1	Ref	1	Ref	1
≥45 years	−0.56 (0.13)	0.95 (0.73–1.22)	0.40 (0.14)	1.04 (0.79–1.37)	0.05 (0.17)	1.06 (0.75–1.48)	−0.45 (0.19)	0.64 (0.44–0.92)*
Sex								
Male	Ref	1	Ref	1	Ref	1	Ref	1
Female	0.14 (0.12)	1.15 (0.91–1.46)	0.23 (0.13)	1.26 (0.97–1.64)	0.09 (0.17)	1.10 (0.80–1.52)	0.37 (0.17)	1.44 (1.03–2.03)*
	Discrimination (c-index) = 0.673 Hosmer–Lemeshow test, *p* = 0.51		Discrimination (c-index) = 0.64 Hosmer–Lemeshow test, *p* = 0.66		Discrimination (c-index) = 0.63 Hosmer–Lemeshow test, *p* = 0.845		Discrimination (c-index) = 0.68 Hosmer–Lemeshow test, *p* = 0.14	

Logistic regression models for the dependent variable of food being purchased in a modern (supermarket or convenience store) retail format rather than a traditional (fresh market) setting. Independent variables are region, urban or rural residence, income, age, and sex. Separate models are presented for four different food types (rice, meat, fresh produce, and dried packaged foods)

# Adjusted odds ratios (AORs) adjusted for age, sex, income, urban/rural residence, and region of residence

* *p* < 0.05; ** *p* < 0.01

### Questionnaires: factors influencing food retail format choice

Mean rankings of importance of various factors when choosing a food retail shopping venue are presented in Table 7. Overall, the most important factor was hygiene or cleanliness of food available. This was followed by having a large variety of food on sale and then the convenience of the store’s location. Least important were the availability of promotions, sales or coupons, and then the availability of local/traditional foods. Comparatively there was some difference in rankings provided by various socio-demographic and shopping pattern groups. Females ranked all factors as more important than did males. The biggest difference was in the importance of local/traditional foods being available which was ranked significantly higher by fresh market shoppers, rural residents, and low-income participants. Hygiene and cleanliness was more important for supermarket shoppers as was the availability of promotions/sales/coupons. The affordability of food was more important for low-income and younger participants.

**Table 7:** Mean scores for importance of store characteristics when choosing shopping venue

	Affordable food	Convenient location	Car parking	Hygiene/clean food	Customer service	Variety of food	Local traditional foods	Physical appearance of store	Promotions/coupons available	Healthy options available
Overall	7.8	8.55	7.88	9.29	7.98	8.84	7.0	7.25	5.51	7.65
Male	7.58***	8.37***	7.92	9.18**	7.85**	8.68***	6.99	7.15*	5.17***	7.39***
Female	7.97***	8.69***	7.88	9.39**	8.08**	8.96***	7.01	7.35*	5.81***	7.86***
Shopping pattern										
Fresh market	7.88	8.53	7.88	9.26*	7.95	8.85	7.23**	7.28	5.21**	7.66
Mixed	7.51	8.50	7.79	9.25	7.88	8.71	7.12	7.19	5.81**	7.71
Supermarket	7.86	8.62	7.97	9.39*	8.10	8.90	6.48**	7.27	5.96**	7.60
Residence										
Rural	7.68	8.45	7.87	9.24	7.90	8.77	7.31**	7.25	5.04**	7.62
Urban	7.85	8.59	7.89	9.32	8.01	8.86	6.89**	7.25	5.70**	7.66
Region										
Bangkok and central	8.01	8.65	7.51	9.31	8.0	8.91	6.56	7.19	5.83	7.66
North	7.82	8.63	8.38	9.17	7.99	8.79	7.43	7.38	5.70	7.60
Northeast	7.50	8.42	8.16	9.29	7.97	8.77	7.25	7.33	5.32	7.62
South	7.54	8.29	8.09	9.44	7.94	8.79	7.46	7.25	4.62	7.77
Income										
Low	8.00**	8.56	7.62**	9.27	8.12**	8.87	7.19**	7.30	5.49	7.74
High	7.59**	8.55	8.18**	9.32	7.82**	8.80	6.82**	7.21	5.57	7.56
Age										
<45	7.99**	8.60	7.82	9.34	8.02	8.84	7.04	7.25	5.80**	7.65
>45	7.41**	8.47	8.02	9.21	7.9	8.82	6.91	7.28	4.99**	7.66

* Difference significant <0.1; ** Difference significant <0.05; *** Difference significant <0.00

### Interviews: perceptions and preferences

Results of in-depth interviews provided some more insight into people’s reasons for choosing, and views towards, particular food retail outlets. Most importantly, almost all interviewees had a wide variety of food retail formats available to them, often within a short distance. Their retail choices were made based on values and preferences not by necessity. Nearly every interviewee commented that in the past a fresh market was almost the only source of food and the main change over time was the substantial growth in variety of food sources. This was seen as generally positive with respondents enjoying different aspects of shopping in different venues.

I live in central Bangkok and I like having a large choice of places to shop. I like that I can go to the fresh market and buy my traditional fruit and vegetables and then go to the supermarket for modern foods.

### Atmosphere, car parking, and physical characteristics

The clean, well-organized atmosphere provided by supermarkets and convenience stores was very attractive to the majority of those interviewed and this was generally in contrast to fresh markets which were described as dirty and hard to walk around, particularly those that were on the street or more exposed to the outside environment. Nevertheless, most people still said that the poorer atmosphere at fresh markets did not deter them from using them as their main source for fresh foods. Many also observed that fresh markets in their areas had improved their premises substantially in recent years with stalls being more organized and often new buildings and infrastructure being constructed.

The need for convenient shopping venues was fairly important for respondents, particularly younger ones. Supermarkets were appreciated as a one-stop shopping venue with sufficient car parking making them easy to visit. Only a few respondents did not have access to cars and for them proximity became more important with mobile vendors and local markets being popular. Air-conditioning was also valued and has been a selling point for other forms of modern retail over the last few decades. Increasingly air-conditioning is seen as a necessity rather than a luxury in hot climates (Isaacs 2009; Isaacs et al. 2010).

The air-conditioning at the supermarket is important. It makes us happier as customers and also keeps the food fresher. (Older male, high income, Bangkok)

I can’t park my car anywhere near the fresh market and that makes it difficult for me. I work a lot and need a convenient place to shop. So I buy everything except fresh food at the supermarket. I go once a week and stock up the fridge. (Young female, high income, Bangkok)

The supermarket is air-conditioned, clean, well laid out, and it is easy to find what you want. The service is good. (Older female, high income, Bangkok)

### Food variety and prices

A major issue for many interviewees was the variety of fresh foods which were available from fresh markets but not from supermarkets. Distinctive Thai vegetable varieties and particularly regional specialties were cited as reasons that people would continue to buy primarily fresh foods from fresh markets. Comments on the issue of foods available at fresh markets included:

I like the special Thai vegetables at the market. You won’t find them in the supermarket. (Older female, high income, Bangkok)

At my local fresh market quite a lot of food is still from home gardens around the area. I like that, they are the types of vegetables you don’t get in modern shops. (Older male, low income, Chiang Mai)

I would never buy fresh food from the supermarket. The quality and freshness is not good enough. They don’t sell any of the real ingredients you need for Thai food. If I want to make coconut milk from scratch I need a fresh coconut, I can’t eat it from a can. (Young female, low income, Bangkok)

The presence of a large number of small shops or stalls was a positive factor in shopping. It was observed that if one did not like the produce on offer from one seller one could keep looking. This was compared with supermarkets, which although they sold more food types, were just one shop and consumers could not compare prices or quality. Many interviewees though then went on to say that would buy other categories of food from supermarkets and convenience stores.

Regarding food prices, the majority of respondents said they thought that fresh produce particularly was cheaper at the fresh market. This was balanced though by the fact that supermarkets often charged more, but for imported, or premium produce (for example, labeled pesticide free). Although respondents had observed prices rising in the fresh markets they visited they found that the competition inherent in the fresh market having a large number of small traders gathered together helped contain price rises:

The vegetables are always cheaper and better quality at the market. And you can choose how much you want to buy, at the supermarket everything is pre-packaged. (Young female, high income, Bangkok)

I think there is a larger variety of produce at the market and with lots of stalls you can pick and choose until you find the right quality and price. (Young male, high income, Bangkok)

### Healthy food availability

A young man from Chiang Mai observed that Thai people were becoming “more health conscious” and that fresh markets were better sources for healthier foods with another young man commenting that:

Everything you need for a healthy diet is in a fresh market, just fruit and vegetables, fresh food. (Young male, low income, Chiang Mai)

In contrast supermarkets provided less healthy foods

I like that the fresh market only sells raw healthy foods and no junk foods and instant foods. (Older female, high income, Bangkok)

One young woman observed that being presented with junk food made it difficult to resist, a key factor in modern retail.

I can probably eat more healthily if I only shop at the fresh market, you don’t get tempted by all the junk food on sale at the supermarket. (Young female, high income, Bangkok)

Thai shoppers observations that supermarkets encourage consumption of less healthy processed foods supports an increasing body of research concerning the role of supermarkets in influencing diets (Hawkes 2008; Gomez and Ricketts 2013).

### Hygiene, cleanliness quality of food

Hygiene and cleanliness are highly valued attributes of food retail; however interviewees had mixed opinions on whether fresh markets or supermarkets had better quality, more hygienic foods. Some people observed that fresh markets got their foods more directly from the source, but noted that supermarket foods were stored for long periods and were often not as fresh. In Chiang Mai particularly some people observed that a lot of the fresh produce in their local markets still came from home gardens and small farms in their local area and were brought directly to the market. Others, although in the minority, however felt that supermarket air-conditioning and the packaging used in stores kept the food fresher in supermarkets. There was some concern expressed in interviews about the chemical and pesticide residues present in fruit and vegetables in Thailand. The availability of organic foods at supermarkets and also at some fresh markets was attractive and an important factor in choosing where to shop. The majority though felt fresh markets provided better fresher produce with less pesticide use than supermarkets despite the hygiene levels at fresh markets being less satisfactory.

### Cultural and social factors

Almost every person interviewed expressed the importance of fresh markets for Thailand culturally. This included the availability of culturally valued foods and markets’ roles in supporting cultural and religious traditions:

I like buying flowers and religious supplies for going to the temple at the fresh market. (Older female, high income, Bangkok)

Others observed that markets represented Thai regional culture more broadly to Thais themselves and to tourists.

I like fresh markets in Chiang Mai a lot. They are a much more interesting place to shop, they help keep our local culture and also they attract tourists. (Young male, low income, Chiang Mai)

Interviewees were aware of the importance of fresh markets for the livelihoods of millions of Thais, and for markets as central institutions in communities (even in urban Bangkok). Fresh markets appear to be less central to Thai community than in the past, but people still felt markets were worth supporting for cultural and community preservation. People considered fresh markets under threat due to competition with modern food retailing.

I think fresh markets are important for Thai culture. If stallholders worked together and pooled their money and worked to improve their premises they would have more chance of surviving. Then we as consumers would get the benefits of supermarkets with nicer atmosphere but can help our poorer community members to survive. (Young male, low income, Bangkok)

Fresh markets give poorer people the chance to make an income. For example they sell a whole fish that people can then clean and cut into smaller portions to sell at their own shops or as mobile vendors. Supermarkets do all this for you. (Older male, low income, Bangkok)

I think Thai communities really value the fresh market. They would like to support them but there is no government interest in helping modernize them. (Young female, high income, Bangkok)

The fresh market stall holders look after their customers more than the supermarket. They try not to put their prices up too much. They are a really important part of local communities. But if they want to keep their market share they may have to clean up more and get air conditioning. But then the prices would go up so who knows what will happen. (Older female, high income, Chiang Mai)

### Past and future of food retailing

Fresh markets were observed to have improved in many ways in recent years partly as a response to the challenges of modern retailing but also because with the growth and globalization of food trade in general a much greater variety of foods are now available at fresh markets. Premises have been improved and made more hygienic in many cases and the Ministry of Public Health conducts regular inspections of interviewees local markets. Most participants felt that there was little chance that fresh markets would disappear in Thailand though some people felt that they would likely continue to decline in market share especially with younger people. The main driver of this change was the increasing need and desire for convenience and lack of spare time. A common observation though was that this did not have to be the case and that if fresh markets continued to evolve and improve their premises and so on that they would continue to attract customers.

I have lived in Nonthaburi all my life and have always gone to the same market. There is a lot more variety of food available there now and they have made the market cleaner and kept the meat and fish in separate sections from the vegetables. Also they are open longer hours than they used to be which is easier for modern lifestyles. (Older female, low income, Bangkok)

Perhaps the greatest requirement is for fresh markets to be cleaner and more modern (and particularly to introduce air-conditioning).

My local market has changed a little bit over the last 20 years, more variety of vegetables and more imported food. I really like some of the newer markets in Bangkok though. They are clean and modern more like supermarkets. (Older female, high income, Bangkok)

One other interesting observation from several respondents was that in their experience fresh markets had regained some popularity in recent years as people become “more interested in local culture after years of wanting to become western” (young male, Chiang Mai). Parallels could perhaps be drawn to the middle-class phenomenon in many western high income countries of “re” discovering farmers markets and wanting to support more sustainable agriculture, local production, and local farming cultures (Dixon et al. 2007; Parkins and Craig 2009).

## Discussion

Here we present the results of a study of Thai consumers and their food retail shopping habits and preferences. We assess whether after more than 15 years of exposure to modern food retailing, consumption patterns are moving towards modern formats and how that movement is mediated by socio-economic status, region of residence, and other factors. Our interpretations are based on Reardon and Berdegue (2006) theory of three axes of retail diffusion (socio-economic, geographic, and product category).

Our study found that nearly half of our sample across age and socio-economic groups regularly access modern food retail formats and less than a quarter never do. There were some differences between groups, however, with Bangkok residents and higher income respondents tending to shop at modern retail outlets more. There was a significant difference in where people bought particular food products. Fresh foods including fruit and vegetables and meat, fish and chicken were predominately bought at fresh markets while dry, packaged, and instant foods were bought primarily at supermarkets and convenience stores. Again though, higher income and urban residents were more likely to buy all food types at supermarkets. This partly reflects relative prices of different food categories, with packaged foods being cheaper at supermarkets and fresh foods cheaper at the fresh market but also perceived differences in quality, variety, and culturally valued food availability.

These results indicate that modern food retailing has a strong position in the Thai food market. Substantial proportions of consumers are familiar with supermarkets and other modern retail formats and utilize them on a regular basis. Although high-income and more urban consumers shopped at modern outlets more regularly, rural and low-income groups and those in regions outside Bangkok also do some of their shopping at these outlets. However, the distribution found in our survey of where consumers were purchasing their fresh foods indicates that there are still some major obstacles to modern retail achieving the high market share across all product categories enjoyed in most high income economies.

Qualitative interviews elicited some of the reasons for these differential buying patterns. Supermarkets were much preferred by many consumers on factors ranging from atmosphere (particularly air conditioning), car parking availability, hygiene, and price (for some food types). When buying fresh foods though these qualities were considered less important than the freshness of produce at the fresh market and the availability of traditional local vegetables and local food stuffs. The importance of fresh markets in local culture and livelihoods also appeared in many responses. Despite a long and substantial exposure to supermarkets (in most cases 20 years or more) there was still little indication that consumers were moving towards or planning to move towards buying their fresh foods in supermarkets. Although Thais are exposed to a modern globalized culture they retain a strong pride in their culinary culture and there is a fundamental link between this local food culture and fresh markets which supermarkets are so far not able to penetrate. Also important are observations that Thai fresh markets are already beginning to adapt and change to modern lifestyles and consumer requirements and preferences. Opening hours are changing, more diverse foods are available and perhaps most importantly physical premises are being improved and made more hygienic. This process is being assisted by government agencies, particularly the Ministry of Public Health, and may bolster the long-term survival of fresh markets.

The expansion of supermarkets in Thailand into the fresh food sector may face cultural and economic limits, at least in the short to middle term. Our study revealed some of the cultural and social reasons behind consumer preferences for fresh markets. According to a recent comprehensive study of the Thai retail sector by Gen (2013) the commercial limits on continued modern retail expansion may be equally important. He observes a “mosaic” retail environment in Thailand with Bangkok and major regional centers already experiencing modern retail saturation and the rest of the country (where over half the population still live) having very low demand for new retail formats. This is largely due to high-income inequality, with very low disposable incomes in rural areas. With such dispersed coverage distribution costs are high and competitive advantage becomes difficult. He concludes, as does our study, that a mixed and diverse food retail environment is the likely outcome (Gen 2013).

Research in Hong Kong and mainland China has also shown that, as in Thailand, modern retail had to some extent achieved diffusion in socio-economic and geographic segments but their failure to capture the fresh food sector was restricting the ability of modern retail to continue to capture market share (Goldman et al. 2002; Goldman and Vanhonacker 2006). Interesting evidence has emerged in Brazil where despite the several decades of exposure to and expansion by large scale modern food retailing market share has not continued to grow, in fact traditional retail and small independent stores have actually expanded their market share in recent years (Monteiro et al. 2012). The future for modern food retailing in developing countries then may not necessarily be continued exponential growth. Control of the fresh food sector may remain elusive and the long run outcome, particularly in East and Southeast Asia, may be a bifurcated food system, with modern and traditional retail each retaining a significant share of the market (Humphrey 2007).

Although our sample is drawn from a primarily urban and relatively well educated segment of the Thai population the conclusions drawn are important for the future of food retailing in Thailand. This group, being urban and largely middle class, has been exposed to supermarkets and other modern food retailing for longer than many others in Thailand and we can therefore see how this long term exposure affects shopping behaviors and attitudes towards various food retail formats and predict future trends in the food sector. As we have observed the continuing dominance of the traditional retail sector in the fresh food purchasing behavior of this sample group has important implications, implying a limited ability of supermarkets to expand market share. This has important health equity implications for the Thai population. Fresh markets act as important livelihood sources for both market sellers and their communities, who would not easily find employment in modern food retail sectors. As well as market vendors themselves, a veritable army of hawkers nationwide, informal workers, rely on cheap wholefoods from fresh markets to further process and add value for their income. Also, poorer Thais rely on fresh market access for affordable health promoting fresh foods (Kelly et al. 2010).

However, in most western developed nations there was a long time lag between the emergence of modern food retail and their eventual domination of the fresh food sector (Reardon et al. 2012). This domination arises through efficient supply chains and contracts with farmers enabling standardized quality produce to become cheaper than traditional supply chains. Supermarkets can then aggressively undersell traditional retail that in turn becomes less financially viable. Thus the cultural and social limitations on modern retail spread in the short to mid-term, as discussed in this paper, may not be sufficient to halt the process of diffusion over the longer term. As a result, the sustained future of fresh markets may depend on public policy support.

Thai governments in recent years have strongly promoted Thai cuisine, domestically and internationally, particularly through their “Kitchen to the World” strategy and fresh markets are important to maintaining this distinctive food culture. This particular cultural and economic strategy combined with strong consumer preferences for fresh markets and their social economic and health benefits justify the use of policy approaches which more actively protect and assist fresh markets. Such assistance may take the form of improvements in physical infrastructure and facilities as well as the active promotion of fresh markets as healthy food sources and as repositories of Thai food culture. Some regulation on the spread of modern retail has begun in Thailand with large-scale retailers facing restrictions on the sites of new stores and opening hours (Gen 2013), but stronger measures will take political will, which may or may not be forthcoming. What is more certain is that the future of the Thai retail sector will have regional effects, and be itself influenced, as integrated trade evolves among ASEAN nations from 2015.
